# Probing the Interplay between Mo Back Contact Layer Deposition Condition and MoSe_2_ Layer Formation at the CIGSe/Mo Hetero-Interface

**DOI:** 10.3390/ma16062497

**Published:** 2023-03-21

**Authors:** Fazliyana ‘Izzati Za’abar, Ahmad Wafi Mahmood Zuhdi, Camellia Doroody, Puvaneswaran Chelvanathan, Yulisa Yusoff, Siti Fazlili Abdullah, Mohd. Shaparuddin Bahrudin, Wan Sabeng Wan Adini, Ibrahim Ahmad, Wan Syakirah Wan Abdullah, Nowshad Amin

**Affiliations:** 1 UNITEN R&D Sdn. Bhd., Universiti Tenaga Nasional (UNITEN), Kajang 43000, Selangor, Malaysia; 2 Institute of Sustainable Energy, Universiti Tenaga Nasional (UNITEN), Kajang 43000, Selangor, Malaysia; 3 Solar Energy Research Institute (SERI), Universiti Kebangsaan Malaysia (UKM), Bangi 43600, Selangor, Malaysia; 4 College of Engineering, Universiti Tenaga Nasional (UNITEN), Kajang 43000, Selangor, Malaysia; 5 TNB Renewables Sdn. Bhd., Blok B, Level 10, TNB Platinum, No. 3, Jalan Bukit Pantai, Bangsar, Kuala Lumpur 59100, Malaysia

**Keywords:** energy, solar cells, CIGSe, molybdenum, DC magnetron sputtering, molybdenum diselenide (MoSe_2_), deposition power

## Abstract

The effect of Mo thin film deposition power in DC sputtering on the formation of a MoSe_2_ interfacial layer grown via the annealing of CIGSe/Mo precursors in an Se-free atmosphere was investigated. A Mo layer was deposited on glass substrates using the DC magnetron sputtering method. Its electrical resistivity, as well as its morphological, structural, and adhesion characteristics, were analyzed regarding the deposition power. In the case of thinner films of about 300 nm deposited at 80 W, smaller grains and a lower volume percentage of grain boundaries were found, compared to 510 nm thick film with larger agglomerates obtained at 140 W DC power. By increasing the deposition power, in contrast, the conductivity of the Mo film significantly improved with lowest sheet resistance of 0.353 Ω/square for the sample deposited at 140 W. Both structural and Raman spectroscopy outputs confirmed the pronounced formation of MoSe_2_, resulting from Mo films with predominant (110) orientated planes. Sputtered Mo films deposited at 140 W power improved Mo crystals and the growth of MoSe_2_ layers with a preferential (103) orientation upon the Se-free annealing. With a more porous Mo surface structure for the sample deposited at higher power, a larger contact area developed between the Mo films and the Se compound was found from the CIGSe film deposited on top of the Mo, favoring the formation of MoSe_2_. The CIGSe/Mo hetero-contact, including the MoSe_2_ layer with controlled thickness, is not Schottky-type, but a favourable ohmic-type, as evaluated by the dark I-V measurement at room temperature (RT). These findings support the significance of regulating the thickness of the unintentional MoSe_2_ layer growth, which is attainable by controlling the Mo deposition power. Furthermore, while the adhesion between the CIGSe absorber layer and the Mo remains intact, the resistance of final devices with the Ni/CIGSe/Mo structure was found to be directly linked to the MoSe_2_ thickness. Consequently, it addresses the importance of MoSe_2_ structural properties for improved CIGSe solar cell performance and stability.

## 1. Introduction

Renewable energy solutions have become increasingly significant over the past two decades as a result of global climate change. Scientists are driven to develop sustainable energy resources in order to control CO_2_ emissions while global energy demand is rising. Solar photovoltaics is among the most viable renewable energy sources. A significant growth in photovoltaic (PV) installations has been recorded in recent years. In 2021, a minimum of 175 GW of PV systems were installed and commissioned, contributing to the total cumulative PV capacity of 940 GW by the end of the year [1]. Despite the dominance of silicon based PV market, copper indium gallium diselenide (Cu(In,Ga)Se_2_ or CIGSe) films owing to their tuneable band gap, high absorption coefficient, and relatively low temperature coefficients emerged as a potential competitor [2]. Among the available thin-film technologies, the first most efficient thin film solar cell is CIGSe with laboratory scaled cell 23.4% and module efficiency of 19.2% [3]. 

In CIGSe-absorber-based heterojunction cells, soda lime glass (SLG) substrate improves device performance by promoting the diffusion of sodium (Na), from SLG into the CIGSe. Controlled Na diffusion improves device performance by lowering donor-type selenium (Se) vacancies, passivating grain boundaries, and generating oxides (Cu-poor state) that reduce surface recombination [4]. In parallel to Na diffusion, the ohmic contact is expected to form with the CIGSe absorber for optimal charge transfer. Chemical inertness and stable adherence are also crucial for the device’s structural integrity [5]. Molybdenum (Mo) is proven to be the optimal material of all the prospective choices as the back contact layer in CIGSe thin films for its thermochemical inertness, excellent conductivity and inadvertently formed advantageous p-MoSe_2_ interface layer [6]. It is essential to note that the acceptor ions density (Na) at the interface of CIGSe/Mo is apparently equivalent to the Na doping level in the CIGSe, and the proportion of Na accumulation is greatly corelated to Mo surface qualities [7]. These characteristics, however, are highly influenced by thin film deposition process factors. In general, molybdenum films can be deposited using DC magnetron sputtering, and several research have studied the best growth conditions for Mo utilising DC sputtering since Scofield’s ground-breaking research [8]. Sputtering process factors including sputtering power, pressure, and working distance are well reported to influence the characteristics of sputter-deposited Mo thin films [9]. For instance, increased deposition power resulted in enhanced crystallites, poor adherence and reduced resistivity of Mo layer. In contrast, reduced pressure allows for better resistivity yet extremely poor structural characteristics [10]. Given the need of stable adhesion and conductive back contact for highly efficient CIGSe thin films, with a single Mo layer is exceedingly challenging. As a result, the goal of this research is to address the impact of DC magnetron sputtering deposition conditions, specifically power variation, on the specifications of monolayer Mo thin film and, ultimately, to develop a suitable Mo thin film single layer with improved properties. Despite extensive study on the effects of sputtering parameters [8,11,12]. Just a few research on RF power optimization and the resulting Mo film qualities are documented in the literature [13]. Accordingly, one of the objectives in this work is to reduce the back contact thickness with single/mono layer of Mo, which is more cost-efficient. Furthermore, a systematic study efforted to strike a trade-off between the quality of the Mo thin film microstructure and the formation of MoSe_2_. 

The formation of a MoSe_2_ interface at Mo/CIGSe has been investigated in other research works and the different growth techniques have been studied in different articles [14,15,16,17,18,19,20,21]. The growth of the MoSe_2_ layer has also been reported before, albeit very different scenarios from one to another research were presented. In general, tailoring the MoSe_2_ either by modifying the CIGSe or the Mo microstructural properties is possible through altering the deposition recipe. If only the SLG adhesion and low resistivity are necessary, the Mo layer can be DC-sputtered as a back contact without the need for calibration. However, Mo samples grown without tuning have a lower likelihood of retaining the desired interfacial kinetics, and will not be capable of producing the p-type MoSe_2_ that is advantageous in CIGSe devices, given the relative deficiencies in the Mo deposition profile and Se reactions kinetic with Mo. Hence, understanding the MoSe_2_ layer properties and effectively controlling its formation are important. This study is deliberately focused on investigating the sputtering power effect on the MoSe_2_ formation during the Mo sputtering, particularly in the Se-free condition. Hence, addressing the electrical and mechanical properties of prepared Mo films, as conditions n determining the characteristics of MoSe_2_ formation between CIGSe and Mo, is an absolute prerequisite.

## 2. Experimental

### 2.1. Materials and Methods

**Figure 1:**
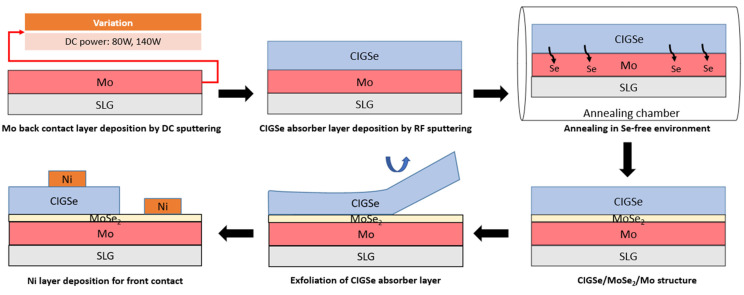
Chronological thin film preparation procedure used in this study.

Mo films were DC sputtered onto 3 × 3 cm^2^ SLG substrates using planar magnetron up-sputtering, resulting in thicknesses in the range of 0.3 to 0.5 μm. The SLG substrates were ultrasonicated in methanol, acetone, and deionised water and dried by nitrogen gas. The source material was a Mo 99.95 percent pure target with 50.8 mm diameter. To eliminate impurities from the target surface, pre-sputtering was performed for 30 min at 50 W power. In order to start deposition under the ideal condition, the base chamber pressure was reduced to 10^−6^ Torr, and chamber pressure was fixed at 5 × 10^−3^ Torr, keeping an argon (Ar) flow rate of 16 SCCM to provide a relatively large mean free path and efficient sputtering rate onto the substrates [22]. The rotation of the sample holder and the spacing between target and the substrate were set at 1 rpm and 100 mm, respectively. The key process variable, the sputtering power, was set at the value of 80 W and 140 W, corresponding to power density of 4 and 7 W/cm^2^. In situ annealing of 400 °C for 60 min was then performed on the sputtered Mo on SLG. Following that, homogeneously sputtered thin films were created with thickness differences of less than 6% across SLG. The CIGSe was then sputtered on the Mo (denoted as CIGSe/Mo) from a quaternary CIGSe target and annealed in a Se-free environment in a quartz tube furnace with a base pressure of 6 × 103 Torr and filled with pure nitrogen (99.9999%). As reported in other study [19], the MoSe_2_ thickness of the selenized samples using temperatures lower than 550 °C is relatively thin, but it increases rapidly with the increase in temperature over 550 °C. Accordingly, heat treatment was applied at 550 °C in an Se-free environment for 30 min in this research. The developed thin-film stack was made up of four main layers as CIGSe/MoSe_2_/Mo/SLG after the heat treatment. The CIGSe/Mo interface was thoroughly examined through the exfoliation of CIGSe layer. The exfoliation process in this research was a simple and cost-effective approach based on the method of [23], which has also been replicated in recent studies [24,25,26,27]. It involves the application of a mechanical force, e.g., the use of adhesive tape, to exfoliate the bulk materials into mono- and multi-layered flakes (Figure 1). This process exploits the weakly bonded nature of the layers between the planes [28]. Since the 2D MoSe_2_ shows weak van der Waals interactions with neighbouring sheets, the cleavage phenomenon was predicted to be similar to as it is described in previous works [29,30]. Following the lift-off, two surfaces can be differentiated side by side, such as the top surface of CIGSe film and exposed Mo back contact side. Finally, to analyse the conductivity and Schottky diode parameters of the back electrode/CIGSe structure, 100 nm thick Ni was deposited as front electrodes by electron beam evaporation on both surfaces. Figure 1 depicts the chronological sample preparation process.

### 2.2. Film Characterization

For all samples, film thickness measurements were conducted using a Bruker Dektak stylus profiler (Bruker, Billerica, MA, USA). Next, the adhesion characteristics of Mo films on SLG were investigated utilising the cross-cutting tape test. This approach provides a rapid and practical distinction between weakly and firmly adhering thin films. Using a blade on coated samples, several grooves were created. A minimum spacing of 1 mm was preserved between each consecutive cut. For each sample, Scotch tape was applied for 1 min. The adhesion quality of the Mo films was compared to a 0 to 5 reference classification scheme of cross-cut adhesion test method (ISO 2409) [31]. Structural specifications and the crystallinity along the Mo and CIGSe/Mo films’ surface were measured by X-ray diffraction (BRUKER aXS-D8, Bruker) with Cu-Kα diffractometer, 0.02 step size and λ = 1.5408 Å. Morphological characteristics of top and cross section shots were captured using CARL ZEISS MERLIN Field Emission Scanning Electron Microscopy (FESEM) (ZEISS, Jena, Germany) operating at 3 kV, with a scan area of 5 μm. Using an NX-10 Park Systems atomic force microscope (AFM) (Gaia Science Pte Ltd., Singapore), the topography was analysed. Electrical parameters were calculated using an Ecopia HMS 3000 Hall Effect system (Ecopia AI, Toronto, ON, Canada). Raman scattering was measured using an Renishaw InVia confocal microscope equipped with grating of 1800 lines/mm. An emission spectrum and laser power of 532 nm and 10 mW, respectively, from a HeCd laser source were employed in this study. Finally, dark I-V curves of Ni/CIGS/Mo samples for different Mo film deposition powers were measured to uncover the contact properties when ambient in nature. 

## 3. Analysis and Discussion

### 3.1. Mo Film Charactristics with Varied DC Deposition Power

Table 1 summarises the film thickness along with the Scotch tape test findings, which demonstrates the low adherence and the high deposition power rate, which are in line with other reports [13,32]. The grooving and adhesivity tests were carried out immediately after the initial sputtering procedure for all Mo films on SLG. Mo films deposited at 80 W (Mo-80) showed good adhesion, with no major signs of film detachment or crack evidence, even after 6 months of deposition for all samples. The adhesion level of Mo films, essential for back contact usage [16,33], is affected by the growth condition of Mo thin films. 

**Figure 2:**
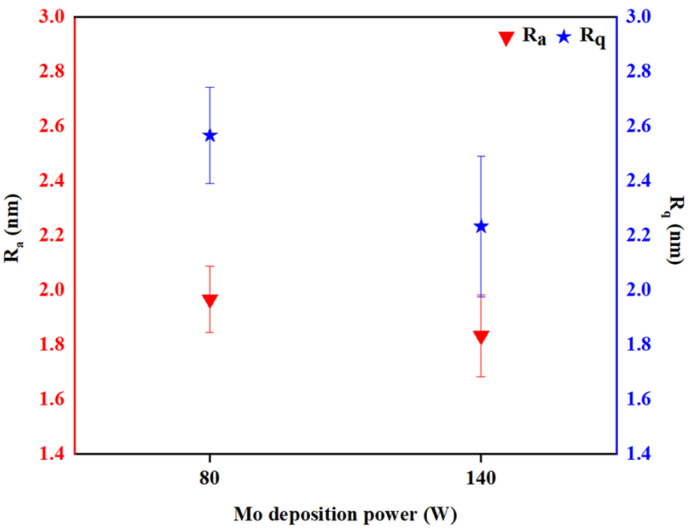
AFM roughness levels R_a_ and R_q_ of Mo-80 and Mo-140 samples, respectively.

Roughness levels of samples deposited at DC power levels of 80 W (Mo-80) and 140 W (Mo-140) are shown in Figure 2 as found from AFM. The acquired data were converted into an image using the XEI program and surface analysis was performed. R_a_ and R_q_, representing average surface roughness and root-mean-square (RMS) roughness, respectively, are crucial aspects to be addressed, where a higher R_a_ and R_q_ value indicates rougher surface. Table 2 reveals a notable decreasing trend in R_a_ from 1.97 to 1.83 nm for Mo-80 to Mo-140, respectively. As expected, a similar observation was recorded for R_q_, reducing from 2.57 to 2.23 nm. The values indicate that in this study, the increasing power rate resulted in a smoother Mo film surface, which is contradictive to the results presented by Rashid et al. [13], which could be attributed to the different process flow involved during the sputtering process. On the contrary, surface skewness (R_sk_) characterizes the symmetry of distribution, where a positive R_sk_ value indicates the peak distribution dominance, while a negative value indicates the surface is valley-dominated [34]. In the Mo-80 sample, many spikes were found in the AFM image, which correlates to a high value of 0.48, in comparison to −1.06 from the Mo-140 sample. 

**Figure 3:**
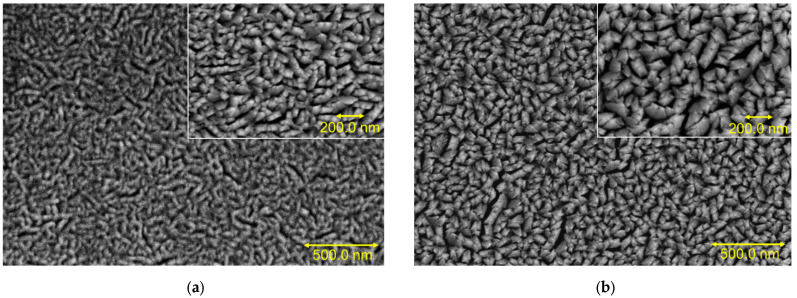
FESEM images of sputtered Mo films at: (**a**) 80 W and (**b**) 140 W.

**Figure 4:**
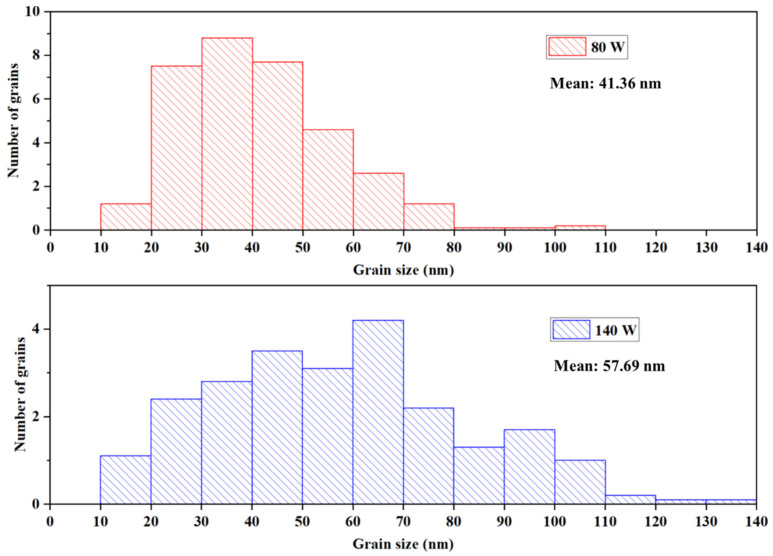
Distribution of grain sizes in Mo-80 and Mo-140 samples.

The FESEM images, shown in Figure 3a, illustrate nano-sized elongated fibrous grains and small pore diameters for Mo-80 film. With the increase in power, the pin holes and fibrous grain size also increased. The Mo-140 sample showed strip structure incorporation, with conglomerates in larger dimensions. Clusters started to form as shown in Figure 3b. The presented results are in line with other studies [35]. According to the literature, increased sputtering power increases grain growth [5]. As a result, a likely interpretation for the grain nucleation and rapid growth using 140 W power rate is the high flux induced to the sputtered atoms. The grains were found to be in the range of 10–140 nm long, as presented in the histogram distribution in Figure 4, with an average grain size of 41.36 nm and 57.69 nm for Mo-80 and Mo-140, respectively. The results indicate that the grain size increases with the power and the surface of the samples changed from rough to compact, validating the roughness analysis from AFM. According to Vink et al. [11], porous microstructures are evidence for lower power, whereas sputtered Mo at a higher deposition power had dense microstructures [13]. With increased power, the grain size increases, which inherently minimises the gap between grains, resulting in the production of densely packed Mo microstructures, as reported in the literature [13,21]. However, interestingly, in this work, the Mo-140 sample shows a more porous microstructure, with distinct voids or inter-grain spaces, which could be due to the deposition condition of Mo, where the in situ annealing of the samples was introduced right after the film deposition.

**Figure 5:**
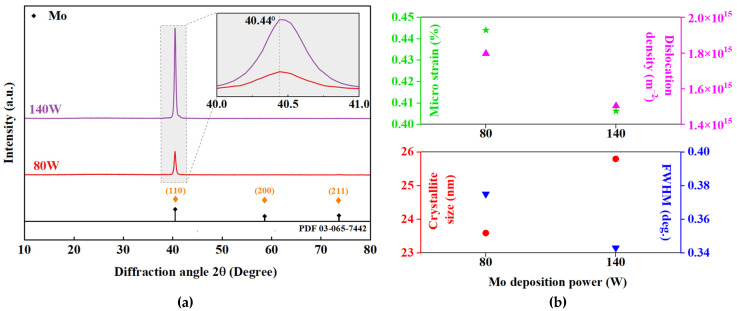
(**a**) XRD patterns of Mo films with sputter power variation and (Inset) XRD peak shift. (**b**) (**Bottom**) Crystallite size and FWHM values (**Top**) Micro strain and lattice parameter.

Figure 5a illustrates the XRD pattern of the deposited Mo samples from 10° to 80°. All films exhibited one main peak, corresponding to the (110) reflection plane at 2θ = 40.44°, indicating monocrystalline features of the sputtered Mo films, which has been reported for low pressure film growth [13]. The obtained data are classified as body-centred cubic (BCC) with JCPDS card No. 65-7442 category. The inset in Figure 5a shows the (110) peak, where no marginal shift of the peak observed with the variation in deposition power. The trend suggests increased peak intensity with power, indicating comparatively strong crystallisation along the favoured orientation for Mo-140. 

Using XRD data, the crystallinity of Mo samples in terms of their lattice configurations and deficiencies were estimated. Structural parameters were calculated using Bragg’s law [$d_{hkl}=\left(\frac{n\lambda }{2\sin\theta }\right)$] while Vegard’s law gives the lattice constant (α) in a cubic structure [$a_{cubic}=d_{hkl}{\left(h^{2}+k^{2}+l^{2}\right)}^{1/2}$, where n, d, and θ represents the positive integer, inter planar spacing, and the diffraction angle of neighboring crystal planes calculated, respectively [36]. The crystallite size ($D_{hkl}$) of the film was extracted from the major (110) peak by using the known Scherrer’s formula [$D_{hkl}=0.9\lambda /\beta \cos\theta$], where β stands for Full Width at Half Max (FWHM) and θ is the Bragg’s angle at (hkl) plane [36]. Micro-strain also occurred as the result of lattice imperfections and distortion, calculated by the Stoke–Wilson formula [ε=β/4tanθ] [37], while Williamson and Smallman’s [$\delta =n/D^{2}$] was used for analysing dislocation density [36]. The calculated parameters are summarized in Table 3. The variation in crystallite size and strain properties extracted from the main (110) peak are also represented in Figure 5b.

**Figure 6:**
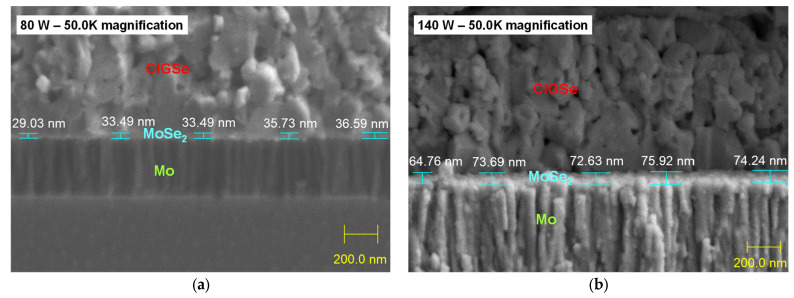
Cross-sectional view of CIGSe/Mo films annealed in Se-free atmosphere for sputtered Mo at different DC powers: (**a**) CIGSe on Mo film deposited at 80 W; (**b**) CIGSe on Mo film deposited at 140 W.

The Mo-80 sample showed a calculated crystallite size of 23.59 nm. Increased deposition power induces grain coalescence, which leads to a larger crystallite size, as seen with a crystallite size of 25.79 nm for Mo-140 film, validating the grain size pattern from FESEM analysis. A lower residual kinetic energy as in 80 W grown films can be translated into smaller crystallite sizes and poorer crystalline quality, leading to a lower surface mobility [38]. In the XRD pattern, FWHM is a measure of dislocation density caused by the ion bombardment during the deposition [39]. Utilising two different deposition powers, the Mo-80 samples yielded a thinner coating, more defects at grain boundaries and substantial stress, resulting in an increased FWHM value of 0.375 in comparison to 0.343 for the Mo-140 samples. The dislocation density was observed to decrease with the increase in power, following the FWHM trend, as presented in Figure 5b, which also portrays an improvement in lattice layout. Furthermore, the metals resistivity is governed by electron scattering, which may take place at various microstructural defect sites, and is totally dependent on grain size and, hence, film thickness. The scattering mechanism is described in [40], where the simultaneous mechanisms of isotropic electron and surface and grain boundary scattering are probable in Mo films. Similarly, here, the scattering phenomenon is probably due to the polycrystalline, columnar grain structure formation, as revealed in the cross-sections shown in Figure 6. The electrical responses of Mo films measured by Hall measurement and outputs are shown in Table 4. 

The values of the aforementioned parameters lie in the range of 10^22^ cm^−3^, 1.6–6.8 cm^2^/Vs, 18–23 μΩ·cm and 0.353–0.747 Ω/square, respectively. A resistivity of around 18 μΩ·cm resulted from Mo-140; meanwhile, sputtered Mo at 80 W demonstrated the highest resistivity, at 23 μΩ·cm. As seen here, resistivity and sheet resistance decrease with the increase in power density, attributed to carrier mobility improvement and relatively constant carrier concentration [22]. In agreement with the results from Gordillo et al., increasing power leads to a decrease in the resistivity, which can be ascribed to the dependency of resistivity on the grain size factor [42]. By comparing conductivity factor with grain size, a complete reverse growth trend is obtained. Increased grain size reduces the grain boundary potential barrier’s height, along with the number of boundaries the carrier has to cross which enhances the conductivity.

### 3.2. Correlation between MoSe_2_ Interfacial Layer Formation and Mo Surface Microstructures

**Figure 7:**
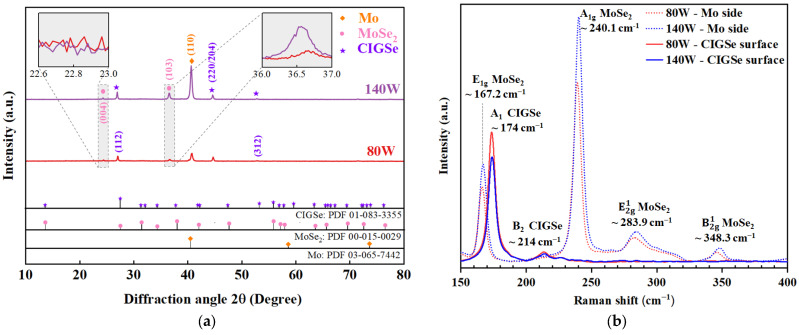
(**a**) 2D view of XRD patterns CIGSe/Mo samples grown at different powers with the presence of MoSe_2_ phase at the CIGSe-Mo interface (**b**) Comparison of Raman spectra from the Mo side (after lift-off) and the CIGSe surface.

The effects of microstructural variations of Mo on the formation of MoSe_2_ is studied here by Mo films deposition at 80 W and 140 W followed by post-annealing treatment in an Se-free environment. Figure 6 shows the cross-sectional view of the sputtered CIGSe/Mo films annealed in Se-free environment. Even though the Mo thin films were deposited at different powers, a cross section view shows similar columnar patterns. A thin MoSe_2_ layer (<100 nm) can be seen at the Mo/CIGSe interface during CIGSe deposition and annealing in Se-free environment for both Mo-80 and Mo-140 samples. Cross-section figures with relative boundaries that split the Mo samples into lighter upper and dimmer bottom halves show a clear growth trend of the MoSe_2_ layer. Clearly, the thickness of the MoSe_2_ phase increases with deposition power, which also increased the thickness of the Mo layer. The cross-section images of CIGSe/MoSe_2_/Mo films shown in Figure 6a,b reveal that increasing the deposition power of Mo film from 80 W to 140 W doubled MoSe_2_ thickness, from the average of 33.67 nm to 72.25 nm. It has been reported in literature that MoSe_2_ thickness has a major effect on the resultant CIGSe solar cells performance [19]. An acceptable thickness for the MoSe_2_ layer (less than 100 nm) allows the formation of a chalcopyrite CIGSe. XRD patterns shown in Figure 7a illustrate CIGSe films annealed in Se-free atmosphere show a chalcopyrite structure (JCPDS number 83-3355). Presented results here also confirm that the MoSe_2_ layer seen in Figure 6 has homogeneous composition and a hexagonal layered structure with a c-axis parallel to the Mo layer [21].

Crystalline intensity of CIGSe phases at 2θ = 27° and 44.6° corresponding to the (112) and (220/204) reflection planes slightly increased when Mo film deposition power was increased, similar to Yoon et al. [43]. The (112) peaks are sharper and no secondary phase detected, which proves highly crystalline oriented for the annealed CIGSe films. As determined by XRD patterns, the presence of MoSe_2_ layer is evident by MoSe_2_ peak recorded in the CIGSe/Mo samples. Characteristics of the MoSe_2_ compound formed at the CIGSe/Mo during CIGSe deposition were portrayed to be identical with MoSe_2_, with hexagonal orientation and lattice constants of a = 0.32870 and c = 1.2925 nm, respectively, that correspond to JCPDS card number of 15-0029 and the (004) and (103) peaks of MoSe_2_ clearly seen in Figure 7a. It was expected that there will be reduction in the metallic-Mo phases (JCPDS card number 65-7442) due to an increase in the MoSe_2_ compounds with the higher Mo discharge power. Nevertheless, the Mo (110) peak remained high for 140 W grown films as compared to the 80 W sample. 

Upon the lift-off, two surfaces can be differentiated from each other for each sample: the top surface of CIGSe absorber layer and the exposed MoSe_2_/Mo side or back contact are as in Figure 1. Raman scattering measurements were performed on the two exposed sides to identify chemical properties and phase formation at the back interface and to confirm the behaviour portrayed by XRD analysis. The samples analysed by Raman spectroscopy were grown using nominally identical process conditions after Mo layer deposition. Figure 7b displays the comparison of Raman spectra measured at both the surface of CIGSe and the exposed MoSe_2_/Mo side of the same samples (Mo-80 and Mo-140). All spectra from the MoSe_2_/Mo side exhibited the most pronounced $A_{1g}$ vibrational mode of MoSe_2_ at around 240.1 cm^−1^ that is related to the vibrations of Se atoms [44]. The other major Raman modes are observed at Raman shifts of 167.2, 283.9 and 348.3 cm^−1^ assigned to the $E_{1g}$, $E_{2g}^{1}$ and $B_{2g}^{1}$ vibrational modes, respectively, of 2H-MoSe_2_ compound, as reported by Sekine et al. [45] conforming the formation of MoSe_2_ layer. In comparison, two key points recorded at the CIGSe surface. First, spectra from the absorber surface are characterized by an intense band at around 174 cm^−1^, assigned with the A_1_ mode of the A^I^B^III^C^VI^_2_ chalcopyrite compounds. In addition to the strong and sharp peak characteristic at 177 cm^−1^, the Raman spectrum displays a minor peak at 214 cm^−1^, which is characteristic of the B_2_ mode of chalcopyrite structure [46]. XRD and Raman spectroscopy analysis also affirmed the succession of the applied exfoliation process, for the purpose of exposing the atomically thin layers of MoSe_2_. As can be observed from Figure 7a,b, no significant peak shift is evident in Raman and XRD peaks for MoSe_2_ indicating that no stress–strain were induced during the removal process. The absence of any Raman peak for CIGSe compound in the signal obtained from the exposed Mo side of Mo-80 and Mo-140 samples confirms the complete removal of CIGSe layer. 

**Figure 8:**
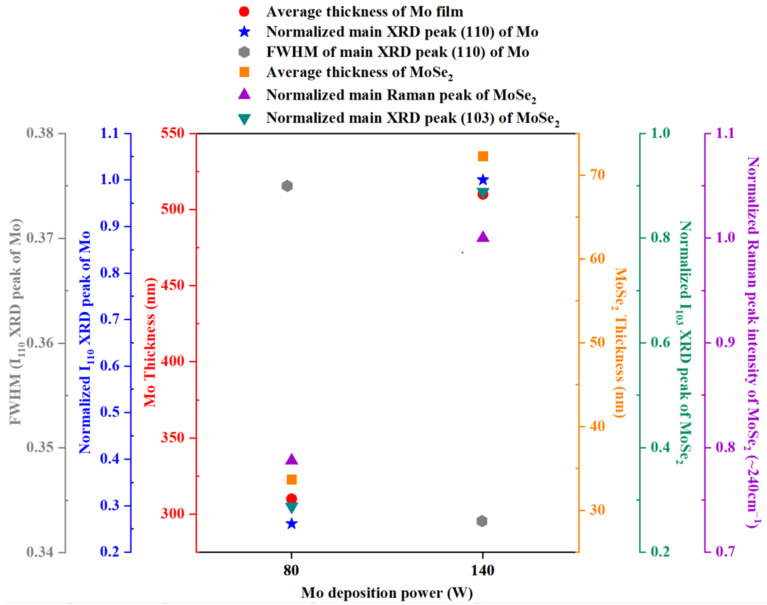
Correlation between A of Mo-80 and Mo-140 samples with B of the corresponding MoSe_2_ film. (A: FWHM values and normalized intensity of (110) XRD peak of Mo; thickness of Mo films; B: MoSe_2_ film thickness; normalized intensity of main (103) XRD peak of MoSe_2_; normalized intensity of main Raman peak of MoSe_2_ compound).

Interestingly, Raman and XRD peaks intensity of MoSe_2_ compound in sample with Mo film deposited at higher DC power is significantly higher than the Mo film deposited at lower discharge power. Consequently, MoSe_2_ thickness growth pattern can also be measured from the XRD trend and Raman characterizations. As shown in Figure 6b, the CIGSe/Mo-140 sample shows thicker MoSe_2_ layer, while in the CIGSe/Mo-80 sample interface thickness is almost halved. Overall, the MoSe_2_ thickness increases with Mo sputtering power. In order to analyse the probable cause of this phenomena, microstructural properties of sputtered Mo films were re-examined in earlier section. Figure 8 shows correlation between the different properties of Mo films deposited at 80 W and 140 W with characteristics of MoSe_2_ compound of the corresponding samples. Evidently, highest Raman characteristic band of MoSe_2_ at 240 cm^−1^ resulted from the sample with Mo film possessing highest peak intensity at (110) plane orientation.

Several models have been developed by other research groups on reaction mechanism of the MoSe_2_ layer and the role of Mo surface microstructure on the growth of MoSe_2_ in CIGSe/Mo samples. In one of the presented models, the interaction between Mo and Se is affected by the Mo morphology, notably the grain density of sputtered Mo, and thinner MoSe_2_ is developed from a denser Mo layer. The Se to Mo ratio seems to be smaller in the denser Mo films [47]. In another suggested scheme, the Mo layer bonding with Se is driven by the residual stress rate in Mo films, and the stronger tensile stress in the Mo film favours forming a thicker MoSe_2_ layer [43]. whereas in another approach, diffused sodium (Na) content from SLG substrate serves as fluxing agent to aid the formation of MoSe_2_, and the thicker MoSe_2_ layer is expected with more Na diffusion [48]. In regards of the fourth model, the sputtering power of Mo determines the contact area between Mo film and Se vapor, attributed to the number of grain boundaries in the film. With the increased power, the formation of MoSe_2_ is suppressed reducing the conversion ratio of MoSe_2_ [21]. The first, second and fourth models are contradictive yet concurrence with our experimental results, while the effect of diffused Na content is not investigated in this study. 

Our findings from morphological, structural, and electrical characterization show remarkably different MoSe_2_ growth behavior from the established models for increasing Mo deposition power. From our discussion in earlier section, direct relation between higher sputtering power with higher quantity of grain boundaries exhibited in Mo films was confirmed, where the grains formed conglomerates with more inter-grain spaces. Therefore, a relatively large Mo/Se contact area from CIGSe film deposited on top of Mo, which could probably increase the reactivity of Mo films with Se and hence, promoting the formation of MoSe_2_ layer at the interface of CIGSe/Mo. Porous microstructure of Mo film at high deposition power yield the swift growth of comparatively thick MoSe_2_ film. Furthermore, it can be deduced that with sputtering power, Mo crystal quality improves, thereby the Mo conductivity increase. The increase in MoSe_2_ film thickness as the sputtering power increases might also be attributed to the fact that sputter power enhances the diffraction intensity and crystallinity of Mo (110) as observed from the pattern shown in Figure 8. The decrement in FWHM value consequently, facilitate the transformation of the cubic crystal structure into a hexagonal crystal structure MoSe_2_ layer [17,19]. The MoSe_2_ layer thickness is also believed to be dependent on the grain density of Mo, and interrelated to the stress and strain in Mo film. The tensile residual stress of Mo in a standard sputtered Mo film significantly reduces with the deposition power value [13]. With the deposition power, the atomic agglomeration across grain boundary increases, creating porosity, thus reducing the tensile stress. It is commonly understood that porous materials possess higher specific surface area and thus increase the photocatalytic activity. This could be correlated to the Mo samples obtained from this study where more porous Mo surface structure for the sample deposited at higher DC power essentially implies more Mo atoms of underlying second layer are exposed, hence increasing the interaction of Se compound with Mo. Nevertheless, it has to be noted that MoSe_2_ growth is based on a reaction of Mo with Se, rather than a deposition of Se atoms or molecules on the Mo surface. Therefore, the Mo surface morphology, roughness and crystal orientation do not significantly influence the MoSe_2_ growth mechanism. Rather, the number of Mo atoms that are available for interaction with Se to form MoSe_2_ compound plays a more vital part. As depicted in Figure 8, the elevated thickness of the MoSe_2_ layer formed at the CIGSe/Mo interface is highly attributed to an increase in Mo film thickness.

**Figure 9:**
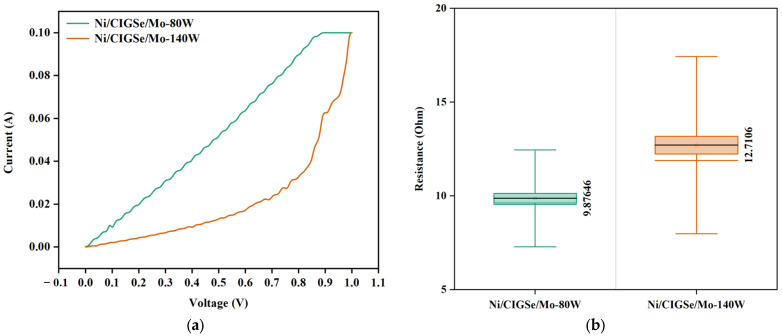
(**a**) Dark I-V characteristic of the device with Ni/CIGSe/Mo structure; (**b**) Stack Resistance.

To further confirm the claim above, dark I-V characteristic curves of the Ni/CIGSe/Mo structures were measured at RT as shown in the following Figure 9a. The dark I-V graph represents a typical diode character of a pn junction and showed that the slope at the forward-current (positive voltage) region was depressed with increasing DC discharge power of Mo from 80 W to 140 W. The diode characteristic of CIGSe/Mo heterocontact presented in this work agrees with earlier works [16,49]. The result suggests that CIGSe/Mo hetero-contact with excessively thick MoSe_2_ layer is no longer ohmic, but rather a Schottky-type contact. With the increase in the thickness of the MoSe_2_ layer from 33.67 nm to 72.25 nm, the resistance of the Mo contact produced at 140 W was higher than that produced at 80 W as shown in Figure 9b, with mean values of 10 Ω and 13 Ω, respectively. In agreement with the observations in previous studies [12,50,51,52,53,54]. Overall, with the reduction in the grain size as the number of grain boundaries reduces, so did the dislocation density and micro-strain, resulting in higher carrier mobility and lower resistivity. The resistance of Ni/CIGSe/Mo structures increased with the increase in Mo deposition power validates our earlier hypothesis regarding the dependence of MoSe_2_ layer formation on the sputtering parameter of Mo, attributed to the semiconducting nature of MoSe_2_ with higher resistivity than that of Mo [13,22,55]. These results imply that growth control of MoSe_2_ layer at the CIGSe/Mo interface could benefit the CIGSe/Mo hetero-contact, changing it from a Schottky to an ohmic-type contact.

## 4. Conclusions

In this study, the microstructural and electrical properties of DC-sputtered Mo monolayer films were investigated for their sputtering powers of 80 W and 140 W. Relatively high sputtering power was demonstrated to increase the grain size, crystallinity, and conductivity in Mo films, while reducing the overall adhesion. Additionally, the effect of sputtering power variation on the formation of MoSe_2_ layer at CIGSE/Mo interface was highlighted. FESEM, XRD, and Raman analyses verified the presence of a MoSe_2_ layer in all CIGSe/Mo samples. The thickness of MoSe_2_ was shown to reach 73 nm, with a Mo sputtering power of 140 W, signifying that the characteristics of sputtered Mo film can aid or impede the qualities of the MoSe_2_ interface. However, 140 W grown samples with a thicker MoSe_2_ layer demonstrated a Schottky contact with a resistance of 13 Ω, indicating that 140 W may be higher than the effective sputtering power necessary for the development of optimal CIGSe solar cells. Ultimately, for a functional CIGSe/Mo hetero-contact, the optimal DC sputtering power range during Mo deposition is between 80 and 100 W.

## Figures and Tables

**Table 1:** Mo sample nomenclature and adhesion results as per ISO standard [31].

Sample Identification	DC Power (W)	DC Power Density (W/cm^2^)	Deposition Time (Minute)	Film Thickness (nm)	Detachment Percentage (%)	ISO Damage Level ^1^	Performance
Mo-80	80	4	60	310	<5% 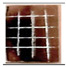	1 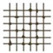	Pass
Mo-140	140	7	60	510	35~65% 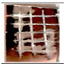	4 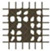	Fail

^1^ Referring to the ISO damage level, 1 indicates very minor detachment of the coating, while 4 refers to major detachment or failure in film adhesion. Black colour indicates the flacked area.

**Table 2:** AFM parameters of Mo thin film with deposition power variation.

Sample	Mo-80	Mo-140
R_a_ (nm)	1.97	1.83
R_q_ (nm)	2.57	2.23
R_q_/R_a_	1.30	1.22
R_sk_	0.48	−1.06

**Table 3:** Calculated structural parameters of sputtered Mo thin films.

Sample	hkl	θ	β (deg)	$a_{cubic}$ (Å)	$d_{hkl}$ (Å)	D (nm)	ε (×10^−3^)	δ (×10^11^)
Mo-80	(110)	20.22	0.375	3.152	0.223	23.59	4.44	1.80
Mo-140	(110)	20.22	0.343	3.152	0.223	25.79	4.06	1.50

**Table 4:** Hall effect measurement results of Mo thin films.

Sample	Carrier Concentration (cm^−3^)	Mobility (cm^2^/Vs)	Resistivity (μΩ·cm)	Sheet Resistance (Ω/square)	Reference Values for Sheet Resistance (Ω/Square) ^1^
Mo-80	5.29 × 10^22^	1.62	23.158	0.747	2.5
Mo-140	5.86 × 10^22^	6.80	17.982	0.353	0.3

^1^ Values taken from [41].

## Data Availability

Not applicable.
